# Angular super-resolution retrieval in small-angle X-ray scattering

**DOI:** 10.1038/s41598-020-73030-2

**Published:** 2020-09-29

**Authors:** Benjamin Gutman, Michael Mrejen, Gil Shabat, Ram Avinery, Yoel Shkolnisky, Roy Beck

**Affiliations:** 1 School of Mathematical Sciences, Tel Aviv University, 69978 Tel Aviv, Israel; 2 The Raymond and Beverly Sackler School of Physics and Astronomy, Tel Aviv University, 69978 Tel Aviv, Israel; 3 The Center for Nanoscience and Nanotechnology, Tel Aviv University, 69978 Tel Aviv, Israel

**Keywords:** Nanoscale materials, Techniques and instrumentation, Mathematics and computing, Scientific data, Techniques and instrumentation, Characterization and analytical techniques, Applied physics

## Abstract

Small-angle X-ray scattering (SAXS) techniques enable convenient nanoscopic characterization for various systems and conditions. Unlike synchrotron-based setups, lab-based SAXS systems intrinsically suffer from lower X-ray flux and limited angular resolution. Here, we develop a two-step retrieval methodology to enhance the angular resolution for given experimental conditions. Using minute hardware additions, we show that translating the X-ray detector in subpixel steps and modifying the incoming beam shape results in a set of 2D scattering images, which is sufficient for super-resolution SAXS retrieval. The technique is verified experimentally to show superior resolution. Such advantages have a direct impact on the ability to resolve finer nanoscopic structures and can be implemented in most existing SAXS apparatuses both using synchrotron- and laboratory-based sources.

## Introduction

The scientific and applicative revolution in nanotechnology requires nanoscale characterization techniques, which are versatile and technologically challenging. Small-angle X-ray scattering (SAXS) is an established technique that has been flourishing in the last decade. Traditionally, SAXS was used to characterize nanoscale objects at low-resolution in various media in a non-destructive way^1–8^. Many SAXS experiments are currently conducted in specialized synchrotron facilities, which harness the high-flux of X-ray photons to provide an adequate scattering signal. Such experiments are not accessible to most industries and scientists, as synchrotron-based experiments are commonly over-booked world-wide.

However, recent advances in small scale laboratory-based X-ray sources and, in particular, in high-efficiency solid-state 2D X-ray detectors, enable conducting many of the experiments using laboratory-based SAXS systems. For example, laboratory-based systems are best suitable for the study of hours-long dynamics for a nanoscopic structural organization^3,9,10^ or when SAXS is used to optimize sample preparation. Currently, there are a handful of well-established SAXS suppliers that provide systems that can measure structural information in a variety of disciplines. Due to limited available time for synchrotron-based SAXS experiments, it is commonly used for cases embracing one (or more) of the synchrotron advantages such as the use of the high X-ray flux, micron-size incoming beam, or variable X-ray energy. Nevertheless, since the pixel size of most X-ray detectors is relatively large (in comparison to the X-ray wavelength), current SAXS systems require significant floor space. A large sample to detector distance (from tens of centrimeters and up to a few meters), typically held in a vacuum (either with or without the detector) is required to achieve high angular resolution in the small-angle region.

For a point source, the SAXS signal, *I*(*q*), measured at a sample to detector distance $d_s$, and at position $\delta r$ from the unscattered direct beam position, is proportional to the Fourier transform squared of the sample’s electron density. for isotropic scattering, $|q| = 4\pi \sin {\theta }/\lambda$ is the modulus of scattering wave vector, $\lambda$ is the X-ray wavelength, and ${2} \theta$ is the scattering angle. From geometrical considerations, $\tan 2\theta =\delta r / d_s$. Therefore, enhanced angular resolution ($\Delta 2 \theta$) requires either large $d_s$ or superior sampling facilitated by a small pixel size of the X-ray camera $\Delta (\delta r)$).

Moreover, similar to conventional imaging techniques, the measured SAXS intensity, $I_m$, is a convolution of the sample scattering intensity with $P(q,\langle q \rangle$), the point-spread function (PSF) of the system, determined by the wavelength spread, finite direct beam collimation, and the resolution of the detector:

1 $$\begin{aligned} I_m(\langle q \rangle )=\int P(q,\langle q \rangle )*I(q)dq. \end{aligned}$$

Here, $\langle q \rangle$ is the average scattering vector corresponding to the setting of the instrument. It is worth noting that for small angles ($q \rightarrow 0$), the collimation-related PSF, which depends on the actual system configuration, can be measured directly from the shape of the direct beam^11^.

From Eq. (1), it is evident that a narrower PSF will yield a better resolution. However, due to limited flux, this requires a significant increase in the time needed for the experiment to achieve a sufficient signal-to-noise ratio (SNR).

Nowadays, PSF deconvolution in SAXS data is performing poorly^12^. In most cases, calculated SAXS models are convoluted with measured or guessed PSFs to fit the experimental data^13^. This procedure is not ideal as it introduces bias into the reconstructed model. Interactive procedures have been proposed to de-smear $I_m(q)$ and obtain the pure scattered intensity data *I*(*q*). However, these methods had limited success in noisy conditions^14^.

Here, we propose a two-step technique that improves the angular resolution. In the first step, hereafter referred to as subpixel sampling (SPS), we acquire the direct beam and the scattered signal with higher resolution than the native sampling resolution (i.e., pixel size) by serially translating the detector in several subpixel steps effectively achieving a smaller pixel size. In the second step, we vary the incoming beam shape, which determines the Point Spread Function (PSF) in both the x and y directions. We then expose the sample to these different beam shapes and apply our Constrained Multi Deconvolution (CMD) algorithm to resolve the de-smeared 2D scattering image. The CMD step improves our angular resolution on top of the SPS, by itself a super-resolution technique. Below we will show the data recording schemes and the analysis schemes as performed on simulated data and on real SAXS experiments. We will further demonstrate that for budgeted experimental time, minor hardware modifications can provide super-resolution SAXS (SrSAXS) capabilities that are useful for probing nanoscale structures using synchrotron and laboratory-based systems.

For the first step, inspired by super-resolution microscopy^15^, we control and monitor the translations of the X-ray detector. We use a sub-pixel retrieval method based on the methodology from Farsiu et al.^16^, and achieve smaller effective pixel size on a commercial 2D X-ray detector. In the second step, motivated by the new solid-state X-ray detectors and the development of scatterless slits design^17^, we control, measure, and analyze the direct beam profile to estimate the blurring PSF. Following, we measure the scattering profiles with different PSFs and retrieve an optimized X-ray diffraction pattern that resolves SAXS data with superior resolution.

Future applications of the suggested advances may have a valuable impact on various aspects and industries as they will provide top-of-the-line nanoscale characterization techniques of quality comparable with that of experiments conducted with superior hardware such as finer detectors or extended path between the sample and the detector. The proposed SrSAXS system allows us to measure smaller scattering angles for a given sample-to-detector distance, or to reduce the sample-to-detector distance for similar small-angle separation resolution and, therefore, significantly improve existing SAXS resolution while enhancing the angular dynamic-range capabilities.

## Results and discussion

In SAXS instruments, a monochromatic beam of X-rays illuminates a sample from which some of the X-rays are scattered, while most go through the sample without interacting with it. The scattered X-rays are collected by a camera (typically a 2D flat X-ray detector) situated behind the sample. However, most available laboratory-based X-ray sources produce divergent beams and thus rely on collimating the direct beam. In our laboratory-based SAXS system, we control the beam shape and flux using the scatterless slits design^17^, allowing the engineering of the system’s response function.

Our super-resolution method is composed of two consecutive phases:(a) subpixel sampling and (b) multi-PSF engineering. Below, we will show that for a budgeted experimental time, a specific experimental protocol results in superior angular resolution. We compare our results to alternative measurement protocols.

### Subpixel sampling (SPS)

In comparison to visible wavelengths, X-ray detectors suffer from relatively large pixel dimensions. For a given experimental setup, this significantly limits the achievable angular resolution, in particular at the smallest angles. To retrieve the scattering with subpixel resolution, we conduct several measurements of a given sample, each time with a different position of the camera. Between consecutive recordings, we translate the detector position with subpixel intervals. For a given pixel size (*l*), we define the resolution enhancement factor (*f*), for which the sub-pixel translation is of *l*/*f*. This results in overlapped pixels in the registered images, where each pixel contains partial information about the higher resolution data.

To extract the high-resolution image, we first find the actual relative displacement between the different recordings using the Lucas-Kanade algorithm^18^, and then apply a super-resolution (SR) algorithm for proper image fusion^16^.

**Figure 1:**
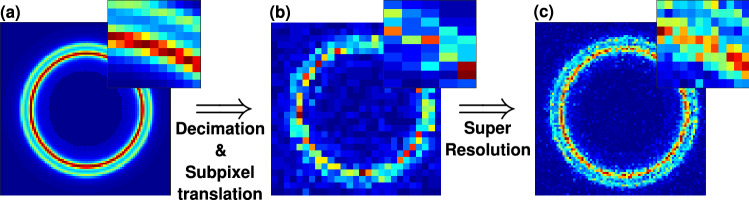
SPS process for a synthetic image: (**a**) A synthetic high resolution ground-truth (GT) of two nearby circular peaks, (**b**) down-sampled translated image of the GT for realistic measurement recording, and (**c**) SPS retrieval. Insets show zoomed-in images.

We applied this approach to synthetic data modeling realistic noise and X-ray diffraction pattern (Fig. 1) with a resolution enhancement factor $f=3$ (9 translations on 3x3 grid). The results demonstrate an improved resolution and our ability to resolve two nearby peaks otherwise undetectable given the original pixel dimension.

Motivated by the synthetic results, we gathered experimental data of X-Ray scattering patterns in a short sample to detector distance ($d_s=117\,$mm). Typically, SAXS is measured with an order of magnitude larger $d_s$. Our test samples are a Silver behenate (AgBh) powder, showing the scattering signal that emanates from a lamellar structure of scatteres, and DOPE phospholipids in solution, showing the scattering signal of a self-assembled inverted hexagonal phase. Both samples exhibit isotropic scattering rings conveniently analyzed in the azimuthally integrated signal with respect to the direct beam center. The samples were measured with $f=3$, $l=172 \, \upmu \hbox {m}$ and with various PSFs.

**Figure 2:**
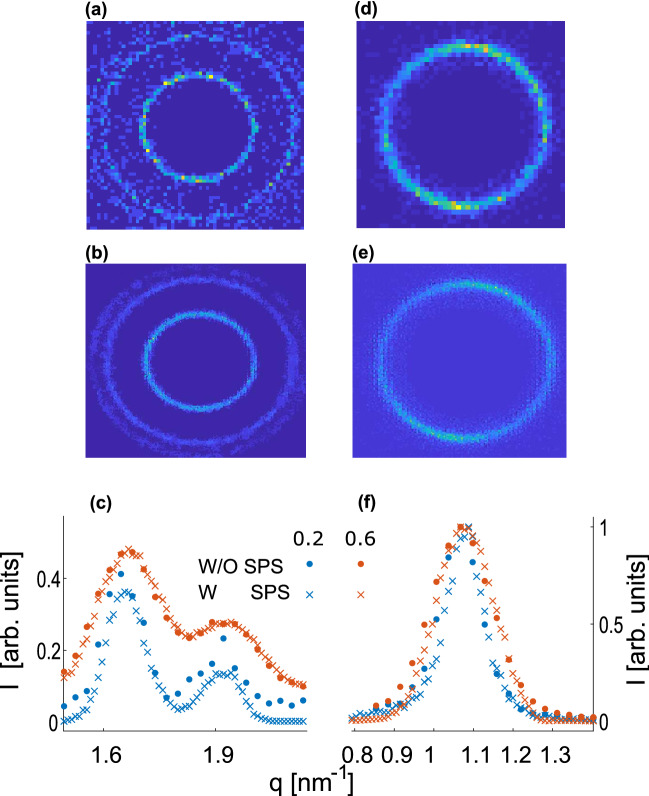
Resolution performance of the SPS algorithm for (**a**–**c**) DOPE and (**d**–**f**) AgBh samples. (**a**,**d**) Measured with $0.2 \times 0.2 \,\hbox {mm}^2$ beam profile. (**b**,**e**) Result of the SPS algorithm with a $0.2 \times 0.2\,\hbox {mm}^2$ beam profile. (**c**,**f**) 1D azimuthal integration SAXS profiles and retrievals with 2 different square beam sizes. For clarity, error bars are presented in the supplementary materials Fig. S4.

Each of the measured samples provides a different perspective of the super-resolution retrieval. AgBh allows investigating the sharpening of scattering features at small angles. On the other hand, the DOPE sample has two close scattering peaks giving a more tangible appreciation of the resolving power improvement of the retrieval algorithm (Fig. 2). We note that many SAXS experiments do not result in Bragg-like diffraction peaks, as with our samples, but rather smooth form-factor features. However, for the latter, quantifying the resolution is intricate. In contrast, we can define a separation criterium of two nearby peaks, such as $q_{H(1,1)}$ and $q_{H(2,0)}$ in the DOPE scattering, by:

2 $$\begin{aligned} \delta =\frac{I_p-I_v}{\Delta q}. \end{aligned}$$

Here, $I_p$ and $I_v$ are the intensities of the lower peak and valley between the peaks, respectively, and $\Delta q$ is the distance between them (see Fig. 2c). We notice that the direct beam profile, and therefore the PSF of the SAXS system has a significant effect on the ability to achieve higher resolution retrievals. For a beam profile of $0.6\times 0.6\,\hbox {mm}^2$ we find $\delta _{0.6\times 0.6} = 0.60$, while for larger beam profile ($1 \times 1\,\hbox {mm}^2$) $\delta _{1 \times 1} = 0$ since no distinction of close peaks is observed. Furthermore, for the AgBh measurements, we find that SPS results in denoising the image and reducing the width of the scattering peak up to $\sim 10\%$.

**Figure 3:**
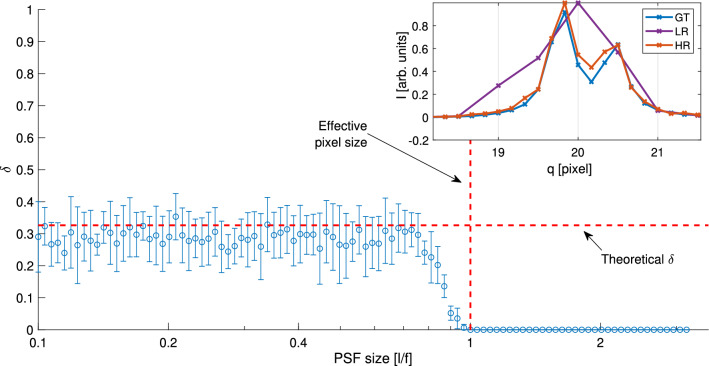
Resolution versus PSF size. Conducting 10 experiments for each PSF size, the circles represent the mean of $\delta$ (Eq. 2) while the error bars stand for the standard deviation. The simulations were conducted using circles at radii 20 and $20 \frac{2}{3}$ pixels, exposure time of 3 (as in Eq. 6) and $f = 3$. The inset represent a typical experiment where LR is the low resolution image (a single exposure), HR for high resolution produced by the SPS algorithm and GT for ground truth. The grid lines (vertical) represent the apparatus pixels.

It should be noted that the SPS algorithm will perform differently on different hardware. Specifically, while the SPS algorithm will yield little to no improvement when the incoming beam (i.e., PSF size) is much wider than the pixel size, the method will yield valuable improvement when the beam width is on the order of pixel size (as shown here) and even better if the beam size is narrower than the pixel size, for example, in synchrotron-based experiments. To demonstrate this effect, we simulated two close circles, located 2/3 pixel size apart. Regardless of exposure time and the PSF size, the scattering profile appears with no separation between neighbouring peaks (See purple line in Fig. 3 inset). Nonetheless, after applying the SPS algorithm the separation is well observed (red line). As expected, repeating the same experiment with different PSF sizes while keeping the exposure time constant, we find that the separating criterion $\delta$ vanishes when the PSF size approaches the effective pixel size (*l*/*f*). Moreover, intuitively, increasing the number of recordings with higher subpixel sampling will yield a higher resolution. However, provided a budgeted time for a given experiment, there is a trade-off between the possible improved resolution and the SNR for each recording. Besides, applying super-resolution methods to a larger translated grid is computationally expensive, with limited benefits in the retrieved 2D image.

The SPS performance also depends on a relation between the actual delta resolution (Eq. 2) and the available PSF size. If the separation between two circles is less than the actual pixel size, any deblurring algorithm will not produce the required separation, regardless of the PSF size and the exposure time. However, an SPS based algorithm may retrieve the actual separation.

### Constrained multi-deconvolution (CMD)

**Figure 4:**
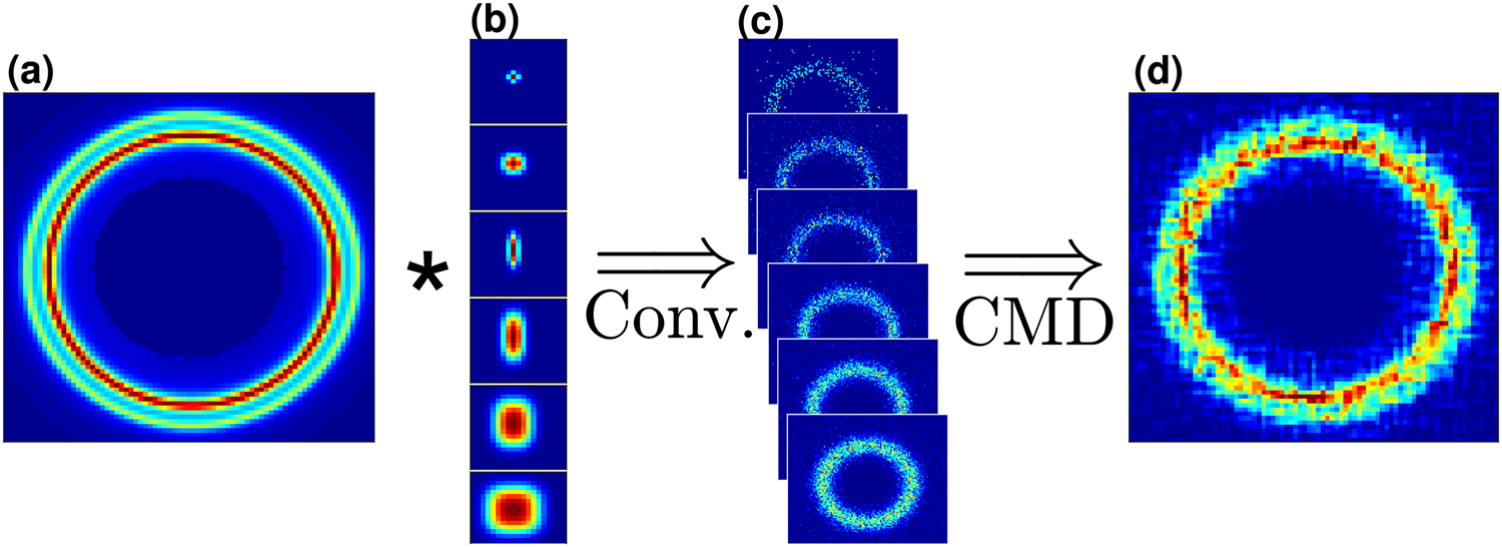
CMD process for synthetic data. (**a**) Synthetic data used to simulate GT, (**b**) synthetic PSF used to blur the GT. (**c**) convoluted images representing blurred measurements. (**d**) The resulting retrieved image after the CMD algorithm.

Another resolution limiting factor is the finite size of the direct X-ray beam. While smaller beams will result in a higher angular resolution, it will impose much longer exposure times to achieve comparable SNRs. Constrained by insufficient flux at smaller beam profiles for laboratory-based SAXS apparatuses, and resolution at larger beam profiles, we develop an algorithm that takes advantage of both. Our new retrieval algorithm, coined as Constrained Multi-Convolution (CMD), is based on measurements with different PSFs of the same sample. For a set of *m* measured images, $Y_i$, and their corresponding measured PSF—$P_i$, where $i=1,\ldots ,m$, our goal is to restore the image as if the PSF was a point source (i.e., delta function). The outcome of such a process will be a de-blurred and de-noised image (Fig. 4).

Formally, we model each measured image as a convolution of the “real” image, X, we wish to restore and a PSF:

3 $$\begin{aligned} Y_{i}=X*P_i. \end{aligned}$$

A direct least squares solution of Eq. (3) can be formulated as following:

4 $$\begin{aligned} {\hat{X}}=\arg \min _{X}\sum _i||Y_i-P_i*X||_F^2, \end{aligned}$$

where $||.||_F$ refers to the Frobenius norm: $||A||_F= \sqrt{\sum _{i, j} A_{i, j}^2}$.

One way of dealing with the resolution to SNR trade-off is phrasing a weight function that controls the significance of each image $Y_i$ in Eq. (4). The weight function should depend on the underlying scattering image, as the following thought experiment demonstrates. For a scattering pattern requiring high resolution (e.g., delta function in q-space), a large PSF will blur the image and, therefore, must not be weighted highly into the algorithm. On the other hand, if the resolution of the available PSF is of the same order as the underlying scattering signal (or finer) then the small PSF images will suffer from low SNR and thus will corrupt the retrieval process. Consequently, a balance between these two extremes is needed when choosing the weights for the CMD algorithm.

We therefore denote by $\sigma$ a weight function and reformulate the CMD retrieval algorithm to:

5 $$\begin{aligned} {\hat{X}} = \arg \min _{X}{\sum _i\frac{||Y_i-P_i*X||_F^2}{2\sigma _i^2}+\nu ||X||_F^2}. \end{aligned}$$

Here, $\sigma _i$ is the weight function for the *i*th PSF, and $\nu ||X||_F^2$ is a ridge regularization required to ensure a numerically stable solution. The regularizing term balances the cost function with the target matrix norm^19^. Future research is still required to obtain an optimal $\sigma$ function, possibly using a machine learning approach. A detailed mathematical formulation of the problem and our proposed solution are summarized in the supplementary materials Section 1.

**Figure 5:**
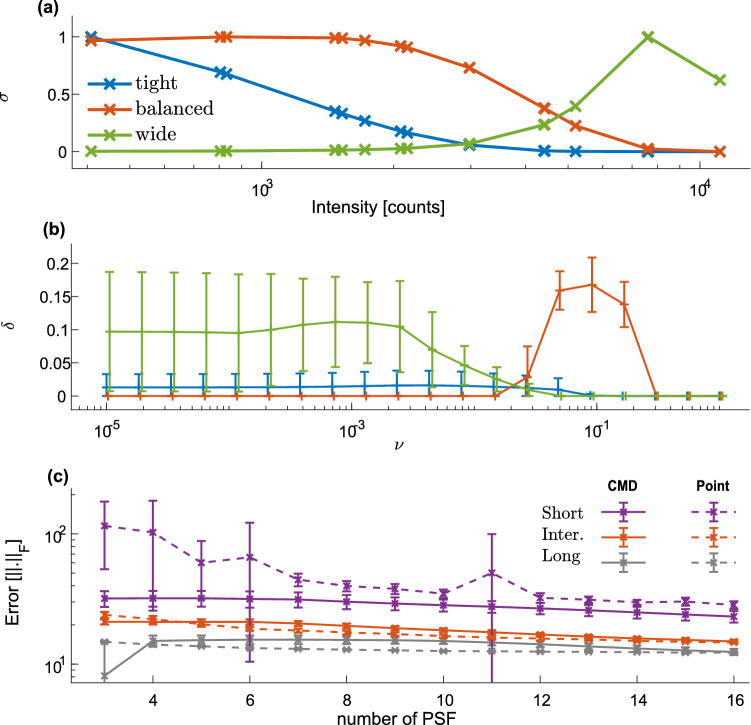
Optimization of retrieval parameters. (**a**) Three different $\sigma$ weight functions were examined for various total intensities in the simulated data: emphasizing tight (blue), balanced (red), and wide (green) PSFs. The total intensity is correlated with the specific PSF area, see supplementary materials Section 2. (**b**) Performance of the CMD algorithm [$\delta$, defined in Eq. (2)] for various regularization factors ($\nu$) and for $\sigma$ weight functions presented in (**a**). (**c**) The error of the retrieval for the CMD algorithm with increasing number of PSFs taken in account (solid lines) using the optimal value of $\nu = 0.1$ and balanced $\sigma$ function (red curve in **a**). Retrieval errors (y-axis) are calculated using the difference between the retrieval and the ground-truth (GT) image (Fig. 4a) in Frobenius norm. For comparison, a single point-like PSF image (labeled as ’point’) with equivalent exposure time is presented in dashed lines. The different lines represent short, intermediate and long exposure times simulation with t = 0.1, 0.8 and 4 respectively, as defined in Eq. (6). The error bars are calculated as the standard deviation from 10 independent simulations and retrievals.

An optimal CMD performance depends on several factors: $\nu$, the duration of the experiment, the number and geometry of PSFs used by the CMD, and $\sigma$ for each PSF. From our experience, while bounds on these parameters can be obtained in general, optimal retrieval parameters depend on the sample under investigation. In the following, we will demonstrate that with optimized parameters, CMD outperforms measurements with the smallest PSF at an equivalent total measurement time.

To evaluate the performance of CMD, we generated synthetic X-ray scattering data with two nearby scattering rings (Fig. 4a) and demonstrated the relation between the retrieval parameters and the resulting resolution (Fig. 5). For three different $\sigma$ functions (Fig. 5a), the resolution of the retrieval has significantly changed with optimal $\nu$ (Fig. 5b). In these examples, for the optimal retrieval, the weights of smaller PSFs are elevated in comparison to the larger ones. Moreover, for a fixed set of PSFs, we find mild dependence between the exposure time (or SNR) and $\nu$ that results in the best retrieval parameters [i.e., larger $\delta$ in Eq. (2)]. Therefore optimizing the CMD performance, in particular for relatively similar SAXS patterns, is feasible.

**Figure 6:**
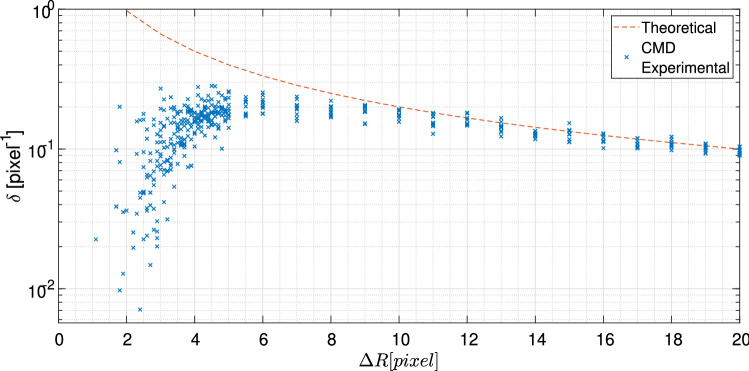
The resolution results of CMD algorithm for separating nearby scattering rings. The separation criteria (Eq. 2), $\delta$, is plotted versus various distances between the rings ($\Delta R$). The red dashed-line is the theoretical value of $\delta$ calculated directly from the GT image.

Furthermore, we evaluated the performance of CMD by altering the separation between two simulated scattering peaks (as described in section "Synthetic data generation"). Here, we fixed the peaks’ width to be 0.7 pixels and one of the rings to a radius of 30 pixels, and varied the radii of the second ring between 31 and 50 pixels (i.e., $\Delta q = 1 - 20$). Using our separation criteria (Eq. 2), we find that the CMD algorithm approaches the theoretical limits (dashed line) for $\Delta q$ larger than 9 pixels, and results in a reasonable retrieval resolution for $\Delta q$ larger than 4 pixels (Fig. 6). Importantly, the deviation from the theoretical $\delta (\Delta q \rightarrow 0)$ is unphysical, since, for a practical system with finite pixel size, all retrieval attempts will fail below the separation of several pixels. Retrieval with various inner circle radii (10, 30, and 50 pixels) resulted in similar conclusions.

**Figure 7:**
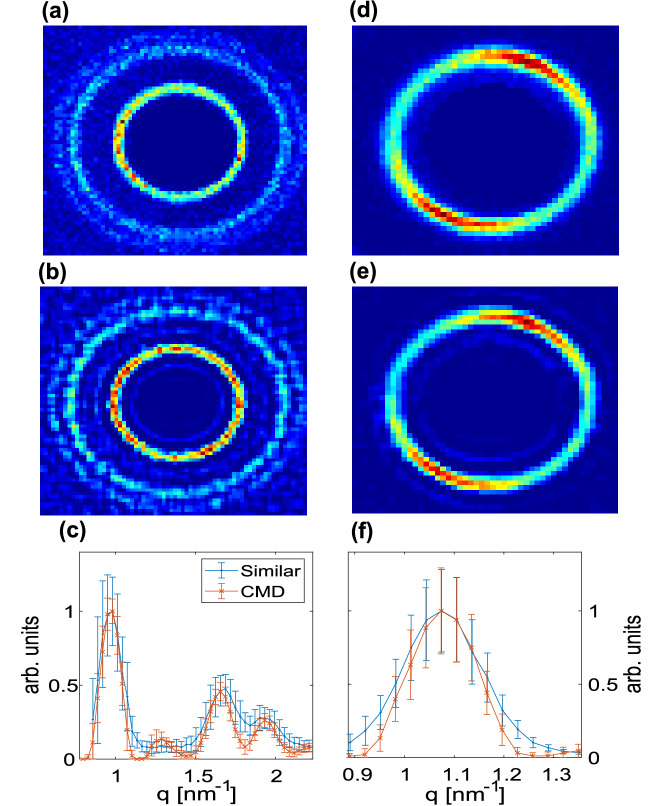
CMD algorithm applied to measured SAXS data: (**a**–**c**) DOPE, (**d**–**f**) AgBh samples. (**a**,**d**) Measurements with PSF size of $0.6\times 0.6~\hbox {mm}^2$ with an exposure time of 3 min. (**b**,**e**) CMD retrieval using 6 different PSFs each with 10 s exposure time. (**c**,**f**) SAXS signal generated by 1D azimuthal integration, presenting enhanced resolution. The minor satellite rings appearing in the reconstruction are discussed in section "Constrained multi-deconvolution (CMD)". Error bars represent the intensity’s standard deviation calculated in the 1D integration.

Last, we examine how the number of different PSFs influences retrieval capabilities. For a given number of PSFs used by the CMD, we evaluated the best performing subset of PSFs (out of a pool of 16 different PSFs). Indeed, increasing the number of measurements results in better retrievals (Fig. 5c). However, increasing the set size comes at the expense of increasing the total measurement time. Thus a reasonable comparison is an equivalent “exposure time” without any PSF blurring (Fig. 5c, dashed lines). Our results clearly demonstrate the supremacy of the CMD retrievals over a large parameter set, and in particular, for short total exposure times, where our retrieval error is significantly smaller than the unrealistic point-like PSF. As exposure time increases, CMD retrieval converge to the optimal point-like PSF, but its benefit diminishes to using only a single small PSF.

Motivated by our simulated data, we proceed to measure the samples mentioned above and evaluating the CMD performance (Fig. 7). In order to optimize the retrieval, we conducted a grid search over the expected $\nu$ and $\sigma$ values of similar patterns studied with synthetic data. We find that the optimal parameters are $\nu =0.04$ and a normal distribution probability function for $\sigma$ centered at 70% and with a width of 20% of the mean intensity over all PSFs used.

The CMD results show noticeable improvement in separation criteria from $$\delta =0.57$$$$\pm 0.23$$ in the measured image (Fig. 7a) to $$\delta =1.15$$$$\pm 0.09$$ in the retrieved image (Fig. 7b). The comparison was done for an equivalent scattering time with the smallest PSF used by the CMD. The retrieval is particularly encouraging since it enables to distinguish between neighboring scattering peaks.

### Super-resolution SAXS Retrieval

We are now in a position to combine the two resolution enhancement approaches described in sections "Subpixel sampling (SPS)" and "Constrained multi-deconvolution (CMD)" to retrieved super-resolution SAXS (SrSAXS) 2D images. The algorithms and synthetic examples are deposited in a public repository^20^. Given that we took *m* images with different PSFs, and we have done so with the resolution enhancement factor *f*, the simplified solution is to perform SPS and CMD procedures separately.

Both from a physical and numerical point of view, the SPS procedure should take place first. Since the blurring arises from the non-point-wise nature of the source, it occurs before the decimation on the camera when the scattered X-ray is stored on finite-sized pixels. Hence, the retrieval should be in the reverse order. From a numerical point of view, performing the CMD first corrupts the sub-pixel translations, and therefore the registration step in the SPS procedure is unable to align the images to one another. This was indeed confirmed in numerical evaluations.

**Figure 8:**
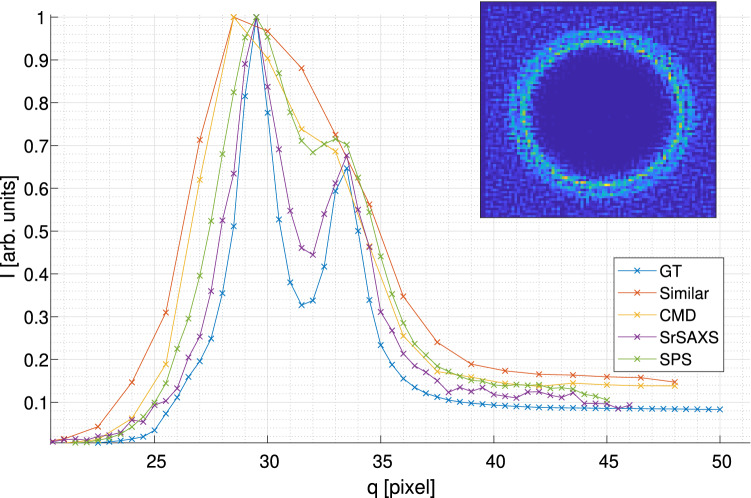
SrSAXS retrieval on simulated data. 1D azimuthal integration of synthetic data simulating two close circles. The addition of the CMD algorithm (purple line) improves the retrieval resolution over the SPS data alone (green line) and even over the SPS data applied on the finest PSF (gray dashed line). 2D retrieval is presented in the inset.“Similar”- images simulated with the smallest PSF and an equivalent total exposure time. The synthetic data was simulated as described in section "Synthetic data generation" with circles of 30 and 34 pixel radii, width of 0.9 and Amplitudes of 1 and 0.6 respectively. For clarity, error bars are presented in the supplementary materials Fig. S5.

In Fig. 8 we demonstrate the added value of the full SrSAXS approach on simulated data having two nearby peaks, which are inseparable at the pixelated blurred image. However, post-processing using the full SrSAXS algorithm, the nearby peaks can be identified with a gain in resolution due to the combined SPS and CMD approach with $\delta _{SrSAXS} = 0.23$. retrieval is superior to the images simulated with the smallest PSF and an equivalent total exposure time used by $\delta _{Similar}=0.031$ (Fig. 8, red dashed line, “Similar”).

**Figure 9:**
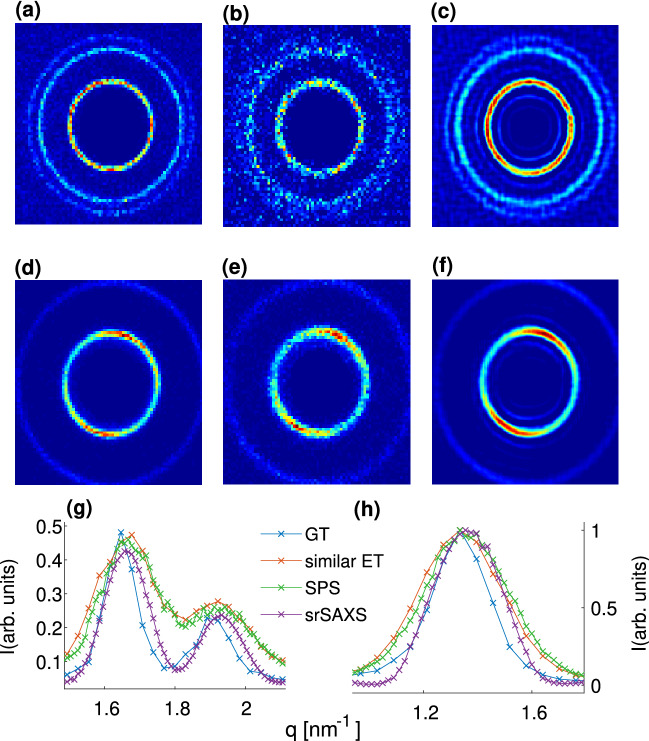
SrSAXS measurements and retrievals for (**a**–**c**,g) DOPE and (**d**–**f**,**h**) AgBh. (**a**,**d**) GT image taken with an exposure time of 15 min and the finest PSF ($0.2 \times 0.2\,\hbox {mm}^2$). (**b**,**e**) Measurements with an exposure time of 10 s and a PSF of ($0.6 \times 0.6\,\hbox {mm}^2$). (**c**,**f**) SrSAXS retrieved images with $\hbox {f}=3$ and 6 different PSFs (detailed in the supplementary materials Section 3). (**g**,**h**) 1D azimuthal integration signals. Blue lines are for the GT as in (**a**,**d**), red lines are measurements of similar exposure times ($9\times 6 \times 10$ s), green lines are SPS retrievals, and purple lines are full SrSAXS retrievals. For clarity, error bars are presented in the supplementary materials Fig. S6.

The combined SrSAXS approach is further applied to our experimental data. In Fig. 9 we present our SrSAXS retrievals for the scattering data of DOPE and AgBh measured at 9 different subpixel positions ($f=3$, i.e. translations of $172/3\,\upmu \hbox {m}$) and using $m=6$ different PSFs. These results manifest the added value of the techniques combined. We do notice that the CMD algorithm produces additional satellite minors peaks. One way of solving this is by choosing other regularization methods, such as L1 or a Tikhonov regularization^19^. Our experiments show different results in this aspect but not with great significance and thus remain for further study.

Evaluating the separation criteria for DOPE (between $$q_{H(1,1)}$$ and $$q_{H(2,0)}$$ peaks) we find $$\delta _{SPS}=0.73 \pm 0.08$$, and $$\delta _{SrSAXS}=1.22$$$$\pm 0.08$$, for SPS only and full SrSAXS retrievals, respectively. In comparison, integrating similar exposure time and only using the smallest PSF used by the CMD ($$0.6 \times 0.6 \,\hbox {mm}^2$$), we find $$\delta _{similar}=0.59$$$$\pm 0.22$$. For the smallest slits opening of $$0.2 \times 0.2 \,\hbox {mm}^2$$, with *twice* the total exposure time we observe $$\delta _{GT}=1.21$$$$\pm 0.12$$, comparable to our retrieval results. Similarly enhanced resolution is also demonstrated for the AgBh data where the (001) peak width shrinks by $$\sim 20\%$$ using our methodology.

**Figure 10:**
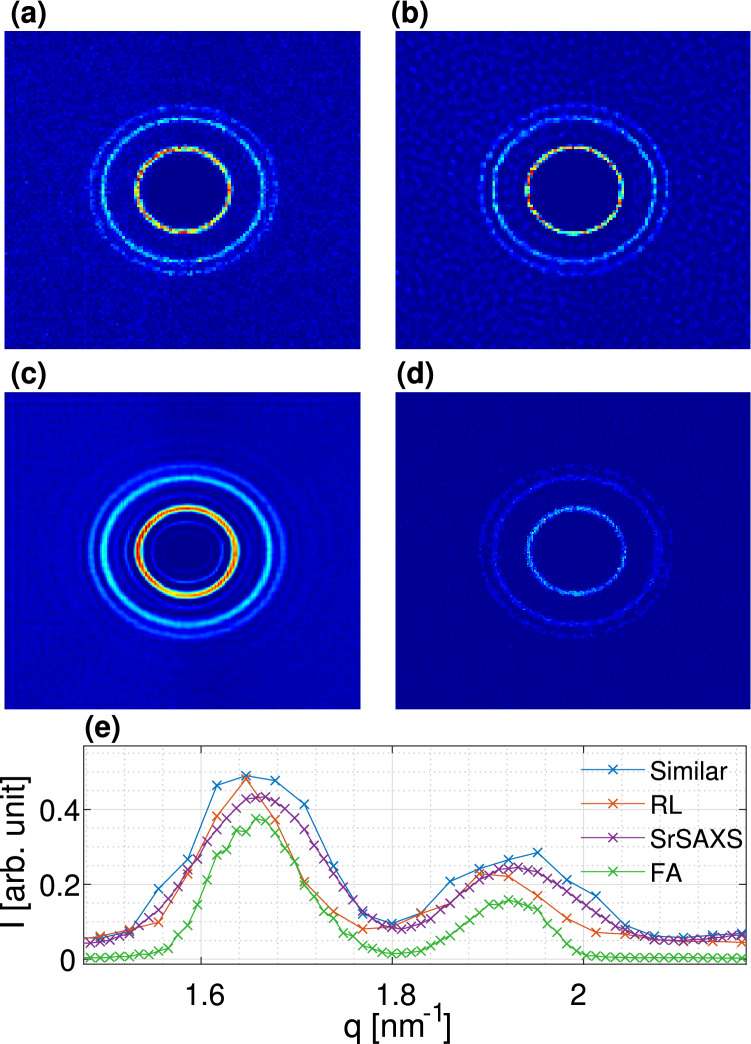
Comparison of different retrieval techniques. (**a**) GT approximation from an exposure time of 15 min with a $0.2 \times 0.2\,\hbox {mm}^2$ PSF, (**b**) Richardson–Lucy deconvolution technique, using the $0.6 \times 0.6\,\hbox {mm}^2$ PSF and 9 min exposure time. (**c**) Our SrSAXS technique with $6 \times 9 \times 10$ seconds total exposure time (as in Fig. 9c). (**d**) Full implementation of Farsiu algorithm (FA)^16^ using the $0.6 \times 0.6\,\hbox {mm}^2$ PSF and $9 \times 1$ min exposure time. (**e**) 1D azimuthal integration. For clarity, error bars are presented in the supplementary materials in Fig. S6.

We compared our SrSAXS algorithms with other retrieval techniques applied to the measured data (DOPE, see Fig. 10). As a common deconvolution technique, we implemented the Richardson-Lucy (RL) algorithm^21,22^ resulting in $$\delta _{RL}=1.19$$$$\pm 0.33$$ (Fig. 10b and red line in Fig. 10e). Additional comparison is presented using the full implementation of the Farsiu et. al algorithm (FA) including the proposed deconvolution^16^ (Fig. 10d and green line in Fig. 10e). The FA results in $$\delta _{FA}=1.17$$$$\pm 0.10$$ with low background signal. Much longer exposure times with a minimal PSF show a very good performance due to high SNRs and negligible blur. However, among the different algorithms examined, our SrSAXS showed better performance in separating nearby features with $$\delta _{SrSAXS}=1.22$$$$\pm 0.08$$ (Fig. 10c and purple line in Fig. 10e).

## Methods

### Synthetic data generation

Three premises are taken in order to generate synthetic scattering images: (a) the ground truth (GT) scattering pattern has circular symmetry with a Lorentzian profile; (b) photon measured by the detector has Poisson statistics; and (c) some spurious background scattering pattern exists, arising regardless of the sample measured, such as scattering of the beam stop, of the sample holder, the direct (unscattered) beam, cosmic radiation, etc. Further details on the background scattering pattern can be found in the supplementary materials Section 4. Summing up the premises outlined above, in order to generate simulated data, we place two close Lorentzian circles, add a background signal that is reciprocal to the distance from the center (i.e., $I_{bkg}\propto q^{-1}$), place a beam-stop for the singularity in the center and run it through a Poisson process:

6 $$\begin{aligned} I[x, y] = poissrnd(t \cdot I_{GT}[x, y]). \end{aligned}$$

Here, *poissrnd* is implemented using Matlab^23^ for random Poisson generator^24^, *t* is the simulated exposure time, and $I_{GT}$ is the ground-truth scattering profile.

For simplicity, we model the PSF as a convolution of a Gaussian source and the rectangular shape of scatterless slits^17^. Since the convolution of a Gaussian and a rectangle is a sum of two error-functions, the PSF is modeled as such. Finally, the different PSFs were convoluted with the ground-truth (GT) image to create synthetic images. Additional details are given in the supplementary materials section 5.

### Samples preparation

Commercial AgBh powder (Thermo Fisher Scientific) was used without any further purification. 1,2-dioleoyl-sn-glycero-3-phosphoethanolamine (DOPE) was purchased from Avanti Polar Lipids Inc. The lipids were dissolved in water (DDW), using a total lipid concentration was 30 mg/ml per sample. The samples were homogenized using a vortexer for 5 minutes at 3000 RPM and were filled in quartz capillaries with a diamter of 1.5 mm, containing about $40\, \upmu \hbox {l}$.

### SAXS measurement setup

Measurements were performed using a lab-based X-ray scattering system, with a GeniX (Xenocs) low divergence Cu $K_{\alpha }$ radiation source (wavelength of $\lambda =1.54$ Å) and a scatterless slits setup^17^. The full-width-half-maximum of the direct beam is 1 mm in diameter, and the measured divergence is 0.0372 degrees ($2 \theta$). Samples were measured at distance of $d_s = 117\,\hbox {mm}$ using Pilatus 300K detector (Dectris) having pixel size of $172\times 172 \, \upmu \hbox {m}^2$ (Ref.^25^). The detector, sample stage, and slits were motorized using stepper motors with a positioning accuracy of 1 $\mu\hbox {m}$ and controlled by SPEC software. In all the measured images, only valid pixels are used. We removed the gaps between the separate detector’s modules in pre-processing. All 2D images presented below were cropped to show the scattering on a single module. The measured flux for the different beam sizes is presented in the supplementary materials Section 2.

### Error estimation

Estimation of the error in the resolution function $\delta$ of Eq. (2), consists of two main contributions: (a) Errors arising from the peak and the valley q values, and (b) errors arising from intensities of the peak and the valley. For the former, the contribution depends only on the effective pixel size (l/f), and therefore $\Delta \delta _{q} \propto \Delta q / q^2$, where $\Delta q$ is the wavevector’s resolution after 1D integration. As for the latter (b), assuming Poisson statistics in both the synthetic and the experimental data, the relative error in estimating the intensity is $I^{-1/2}$, and so for the high SNR region, this contribution can be neglected while for the low SNR region, it can be significant. Throughout the paper, in order to estimate the error in calculating $\delta$, we conduct the same experiment 40 times, allocated the peak and valley using *findpeaks* function implemented in Matlab^23^ and use the standard deviation of the calculated $\delta$’s for the error.

## Summary

**Figure 11:**
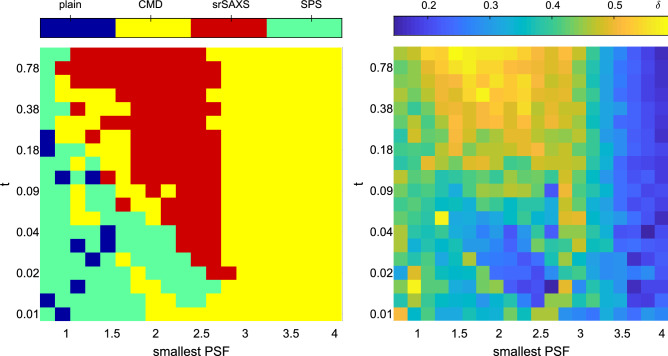
Preferred algorithm for different available PSFs and exposure times. (**a**) Color-coded map of preferred method with largest $\delta$. (**b**) Best resolution function ($\delta$) found by the best algorithm as in panel (**a**). Exposure times (*t*) are given in Eq. (6).

The smallest PSF available and the exposure time (or, equivalently, the SNR) are two features with great diversity in the real-world SAXS devices and experiments. Therefore, it is expected that alternative retrieval approaches should be used to meet the ultimate resolution. For example, in Fig. 11, we compare four alternatives for SAXS retrieval with identical total exposure time: SPS, CMD , SrSAXS (the combined SPS and CMD approach), and a traditional single PSF without any detector translation, which we name plain. For a given exposure time (*t*) and minimal PSF size the following simulations are recorded: 6 different PSFs were simulated from the given smallest PSF (squared) and 2 more sizes: smallest + 1 and smallest + 2 [*l*/*f*]. The resolution enhancement factor, *f*, was set to be 3. A set of images was simulated: a single images for long exposure ($54\cdot t)$, 9 images for the SRS with exposure time of $6 \cdot t$ for each PSF, 6 images with exposure time of $9 \cdot ~ t$ for the CMD, and 54 images (9 for each PSF) for the SrSAXS. The experiment was conducted for 2 circles located radii of 5 and 7 pixels.

In all cases, we assume that the time taking to translate the detector stage (for the SPS approach) or to alter the PSF (for the CMD approach) is insignificant in comparison to the exposure time (*t*). Indeed for large PSFs, the SPS step in unnecessary, and the CMD algorithm dominates all the others in reteriving the data with superior resolution. If smaller PSF’s can be used, as for example in synchrotron-based experiments, SPS step is almost always recommended. For high *t* (i.e., high SNR), the combined algorithms (CMD+SPS) will gain better resolution. While the exact the boundaries between the superior approach can change based on the scattering pattern under study, the above-mentioned statements still holds generally.

We demonstrate a new computationally efficient method that significantly enhances the angular resolution of SAXS experiments. For a limited photon flux, as in the case of lab-based systems, and a limited total experimental time, the recorded SAXS resolution is limited by low SNRs. We demonstrate, both on synthetic and experimentally measured data, two resolution enhancement procedures. In the first method, super-resolution is achieved by measuring the scattering signal for altered sub-pixel positions of the detector. For the second method, several exposures are taken using different PSFs. Each of the techniques resulted in enhanced resolution, while the best performing retrieval is achieved when both techniques are applied one after the other.

## Supplementary information

Supplementary Information 1
