# Supplementary material for: Angular super-resolution retrieval in small-angle X-ray scattering
Source: Sci Rep. 2020 Sep 29;10:16038. doi: 10.1038/s41598-020-73030-2 (PMC7525553; doi:10.1038/s41598-020-73030-2)
Supplement: Supplementary file 1 — Supplementary Information 1 [file 41598_2020_73030_MOESM1_ESM.pdf]

Angular Super-Resolution Retrieval in Small-Angle X-ray Scattering

Benjamin Gutman[1,3], Michael Mrejen[2,3], Gil Shabat[1], Ram Avinery[2,3], Yoel Shkolnisky[1], and Roy Beck[2,3,*]

[1]School of Mathematical Sciences, Tel Aviv University, Tel Aviv 69978, Israel
[2]The Raymond and Beverly Sackler School of Physics and Astronomy, Tel Aviv University, Tel Aviv 69978, Israel
[3]The Center for Nanoscience and Nanotechnology, Tel Aviv University, Tel Aviv 69978, Israel
[*]roy@tauex.tau.ac.il

1 Conjugate gradients details and mathematical proof

Representing the convolution by the corresponding matrix multiplication, Eq. 5 can be solved as a linear system. However, the corresponding matrices are much larger than the actual image, which results in a heavy processing and computational load. For example, convolution of $n \times n$ sized images with an $m \times m$ sized PSF transforms to an $(m + n - 1) \times (n^2)$ matrix. In order to alleviate the computational load, let us rephrase Eq. 5 in operator notation.

For a set of measured PSFs, we define $\mathcal{P}$ to be an operator acting on images by $\mathcal{P} = [P_1* \quad P_2* \quad ... \quad P_m* \quad \sqrt{\nu}I]^T$. In a similar way, we stack the measured images as $\mathcal{Y} = [Y_1 \quad Y_2 \quad ... \quad Y_m \quad 0]^T$. In this notation, Eq. 5 becomes

$$f(X) = ||D(\mathcal{P}X - \mathcal{Y})||_F^2 = \left\| D \begin{pmatrix} P_1 * X & -Y_1 \\ P_2 * X & -Y_2 \\ \vdots & \vdots \\ P_m * X & -Y_m \\ \sqrt{\nu}I & 0 \end{pmatrix} \right\|_F^2 . \tag{S1}$$

Here, D is a diagonal matrix representing σ from Eq. 5:

$$D = \mathrm{diag}(\sigma_1 I \quad \sigma_2 I \quad ... \quad \sigma_m I \quad I). \tag{S2}$$

Differentiating Eq. S1 with respect to X we get the normal equations

$$\bigtriangledown f = (D\mathcal{P})^T D(\mathcal{P}X - \mathcal{Y}), \tag{S3}$$

where $\mathcal{P}^T$ is the conjugate operator in the Frobenius inner product notation. Requiring $\bigtriangledown f = 0$ and using the diagonal form of D, we find our optimum in the form of:

$$\mathcal{P}^T D^2 \mathcal{P} X = \mathcal{P}^T D^2 \mathcal{Y} \tag{S4}$$

This equation is solved using the conjugate gradients method.
Below, we find $\mathcal{P}^T$.

Definition 1.1. Let $A \in \mathbb{R}^{n\times m}$, then $[\breve{A}]_{i,j} := [A]_{n-i,m-j}$

Lemma 1.1. *Defining* $\mathcal{P} = [P_1 * \quad P_2 * \quad \dots \quad P_m * \quad \sqrt{\nu} I]^T$, $\mathcal{P}^T$ *takes the form of* $\mathcal{P}^T \mathcal{Y} =: \mathcal{Y}^T * \breve{\mathcal{P}}$

Proof. We prove the above for $m = 1$ and the extension to a general m is trivial. Recalling that $||X||_F^2 = \langle X, X\rangle_F = Tr(X^T X) = \sum_i [X^T X]_{ii}$, we need to show that $\sum_i [(\mathcal{P}X)^T \mathcal{Y}]_{ii} = \langle \mathcal{P}X, \mathcal{Y}\rangle_F = \langle X, \mathcal{P}^T \mathcal{Y}\rangle_F = \sum_i [X^T(\mathcal{Y}^T * \breve{\mathcal{P}})]_{ii}$.

Therefore,

$\langle \mathcal{P}X, \mathcal{Y}\rangle_F = \sum_i [(\mathcal{P}X)^T \mathcal{Y}]_{ii} = \sum_i (\sum_j [\mathcal{P}X]_{ij}^T Y_{ji}) = \sum_i (\sum_j [P * X]_{ij}^T Y_{ji}) = \sum_{ij} [P * X]_{ji} Y_{ji} =$
$= \sum_{ij} [\sum_{kl} P_{k-j,l-i} X_{kl}] Y_{ji} = \sum_{ij} \sum_{kl} P_{k-j,l-i} Y_{ji} X_{kl} = \sum_{kl} \sum_{ij} \breve{P}_{j-k,i-l} Y_{ji} X_{kl} =$
$= \sum_{kl} X_{kl} [\sum_{ij} \breve{P}_{j-k,i-l} Y_{ji}] = \sum_{kl} X_{kl} [\breve{P} * Y]_{kl} = \sum_l (\sum_k [X_{lk}]^T [Y * \breve{P}]_{kl}) = \sum_l [X^T(\mathcal{Y} * \breve{\mathcal{P}})]_{ll} = \langle X, \mathcal{Y}^T * \breve{\mathcal{P}}\rangle_F$

□

Applying the above in a standard conjugate gradients algorithm finds the optimal X in Eq. S4.

2 PSF measured flux

The PSF measured flux is proportional, approximately, to the aperture area irradiating the sample. In Table S1 we present the values of incoming flux as measured directly on the detector.

PSF $[mm \times mm]$	Area $[\times 10^{-2}\, mm^2]$	Flux $[\times 10^6\, counts/sec]$
0.2×0.2	4	0.258
0.4×0.4	16	2.807
0.2×0.8	16	2.185
0.6×0.4	24	4.959
0.4×0.6	24	5.006
0.8×0.4	32	6.840
0.4×0.8	32	7.137
0.6×0.6	36	9.013
1.0×0.4	40	8.577
0.4×1.0	40	9.705
0.6×0.8	48	12.625
0.8×0.6	48	13.360
0.6×1.0	60	16.295
0.8×0.8	64	18.693
0.8×1.0	80	25.248
1.0×1.0	100	28.799

3 Measured PSF

Conducting measurements included measuring the AgBh and DOPE samples as well as the PSFs used for these measurements. Like the samples, the PSFs were measured 9 times with translations in 2 axes (f=3). The PSFs were determined by the following slit size (measured in mm^2) :
0.2×0.2 ; 0.6×0.6 ; 06×0.8 ; 0.6×1 0.8×0.6 ; 08×0.8 ; 0.8×1

For each sample, we measured an approximation to the ground-truth using the finest PSF (0.2×0.2) with longer exposure times (15 minutes). The rest of the scattering images were measured for 10 seconds intervals.

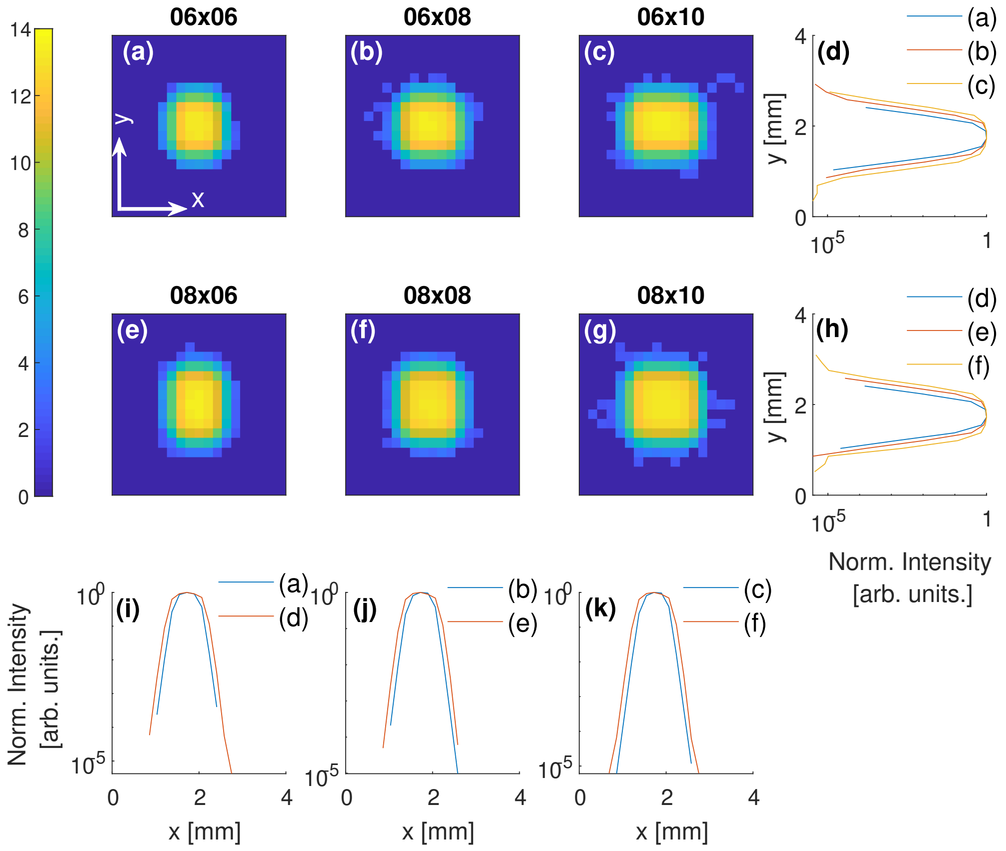

Figure S1: Measured PSFs. Panels (a - c) and (e - g) refer to PSFs 0.6×0.6 ; 0.6×0.8 ; 0.6×1 0.8×0.6 ; 0.8×0.8 ; $0.8 \times 1\, mm \times mm$ respectively, presented on a log scale. (d,h,i-k) Normalized 1D beam profiles or the corresponding PSFs in the same row/column. The PSFs are normalised to their own maximum intensity to demonstrate the beam shaping effect.

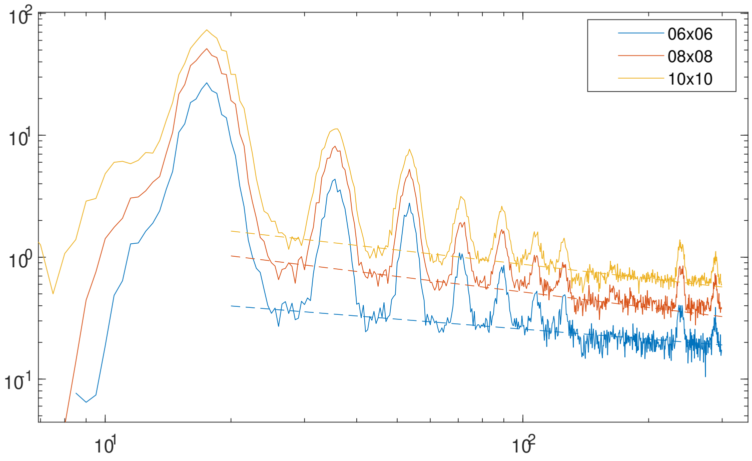

Figure S2: 1D integration of measured AgBh using different PSFs ($0.6\times0.6, 0.8\times0.8$ and $1\times1mm^2$). The dashed lines represent the fitted background signal with $A = 0.99, 2.60, 5.30$ and $B = 0.29, 0.33,$ 0.39, respectively.

4 Synthetic Background signal

From multiple experiments we found that the background signal takes the form of $I_{BKG} = Aq^{-B}$ (Fig. S2). Therefore, in our synthetic data simulation, we used this form of additive background function. Choosing alternative parameters for A and B didn't qualitatively change the reconstruction capabilities.

5 Synthetic PSF

As described in Sec.3.1 the blurring PSFs were modeled with two error-functions at each axis:

$$f(x) = erf(\frac{a_1 + x}{a_2}) + erf(\frac{a_1 - x}{a_2}), \tag{S5}$$

where a_1 is a width parameter, a_2 is the 'sharpness' parameter and x belongs to a symmetrical interval around 0. This 'beam profile' was applied in the x and y axes independently to produce 2D PSFs. Throughout the paper, four such profiles were simulated, resulting in 16 different PSFs, which can be found in Fig S3. The parameters used to generate these PSFs are summarized in Table S1, with $x \in [-8, 8]$ equaly spaced with 19 points.

a_1	a_2
0.1	1
1.6	1.5
3.1	2
4.6	2.5

Table S1: Parameters used to generate synthetic PSFs using Eq. S5

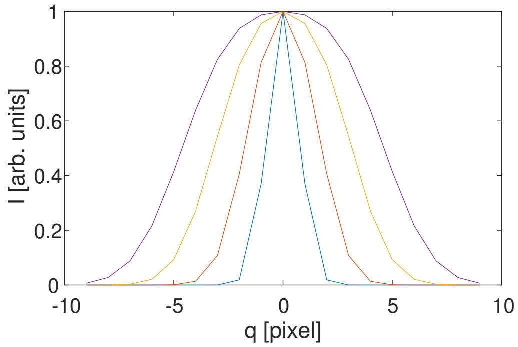

Figure S3: Different profiles used for generating synthetic PSFs

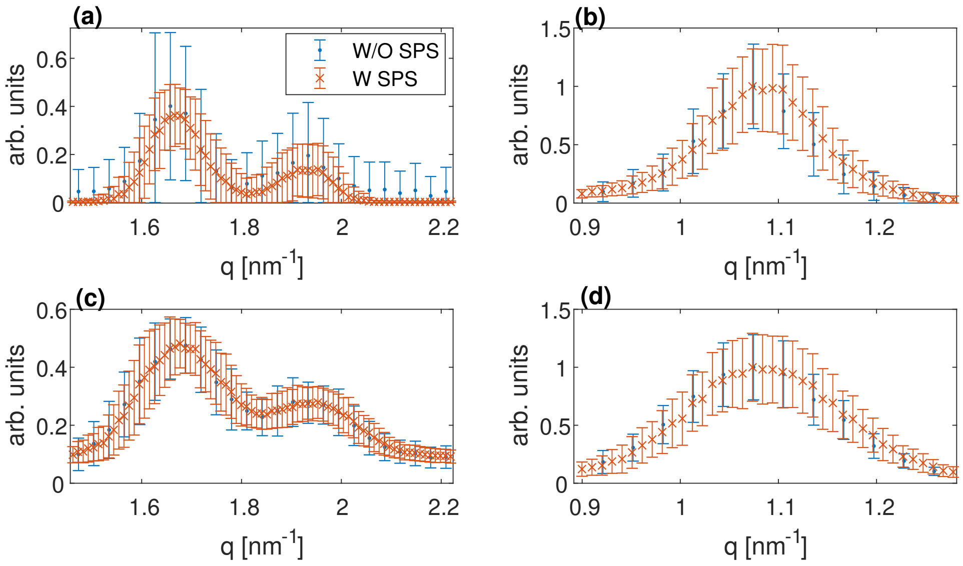

Figure S4: Re-plotting Fig. 2(c,f) with error bar. Error bars are calculated from the intensity standard deviation at the 1D integration, with and without SPS step: (a) result after using $0.2 \times 0.2\ mm^2$ PSF on DOPE sample, (b) $0.2 \times 0.2\ mm^2$ on AgBh , (c) $0.6 \times 0.6\ mm^2$ on DOPE and (d) $0.6 \times 0.6\ mm^2$ on AgBh sample.

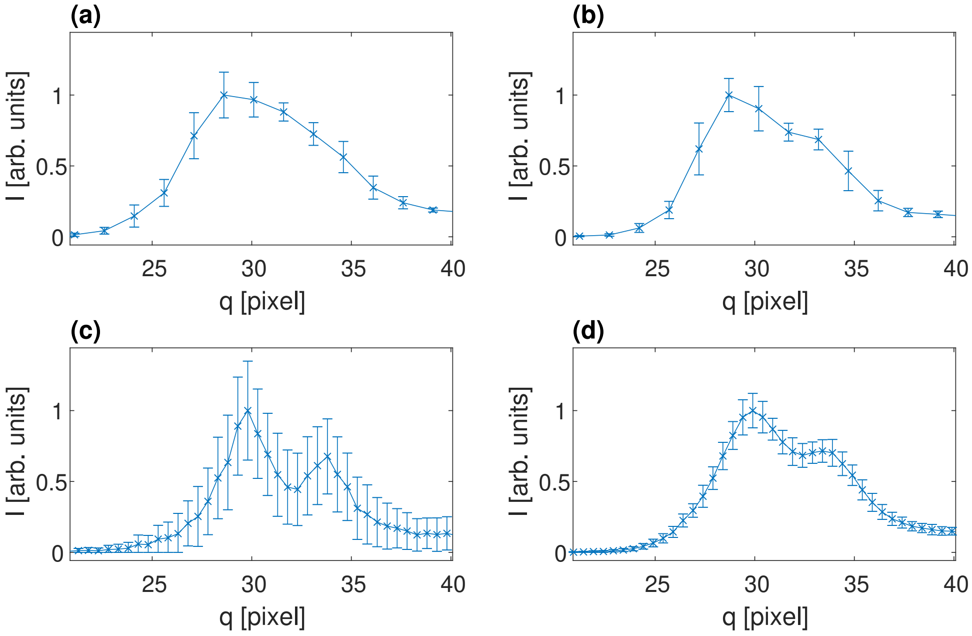

Figure S5: Re-plotting Fig. 8 with error bar. Error bars are calculated from the intensity standard deviation at the 1D integration. (a) long time exposure (similar to the overall time required for the SrSAXS procedure), (b) CMD algorithm using 6 PSFs as specified in section 5, (c) SrSAXS and (d) SPS with f=3. The synthetic data was simulated as described in sec. 3.1 with circles of 30 and 34 pixel radii, width of 0.9 and Amplitudes of 1 and 0.6 respectively.

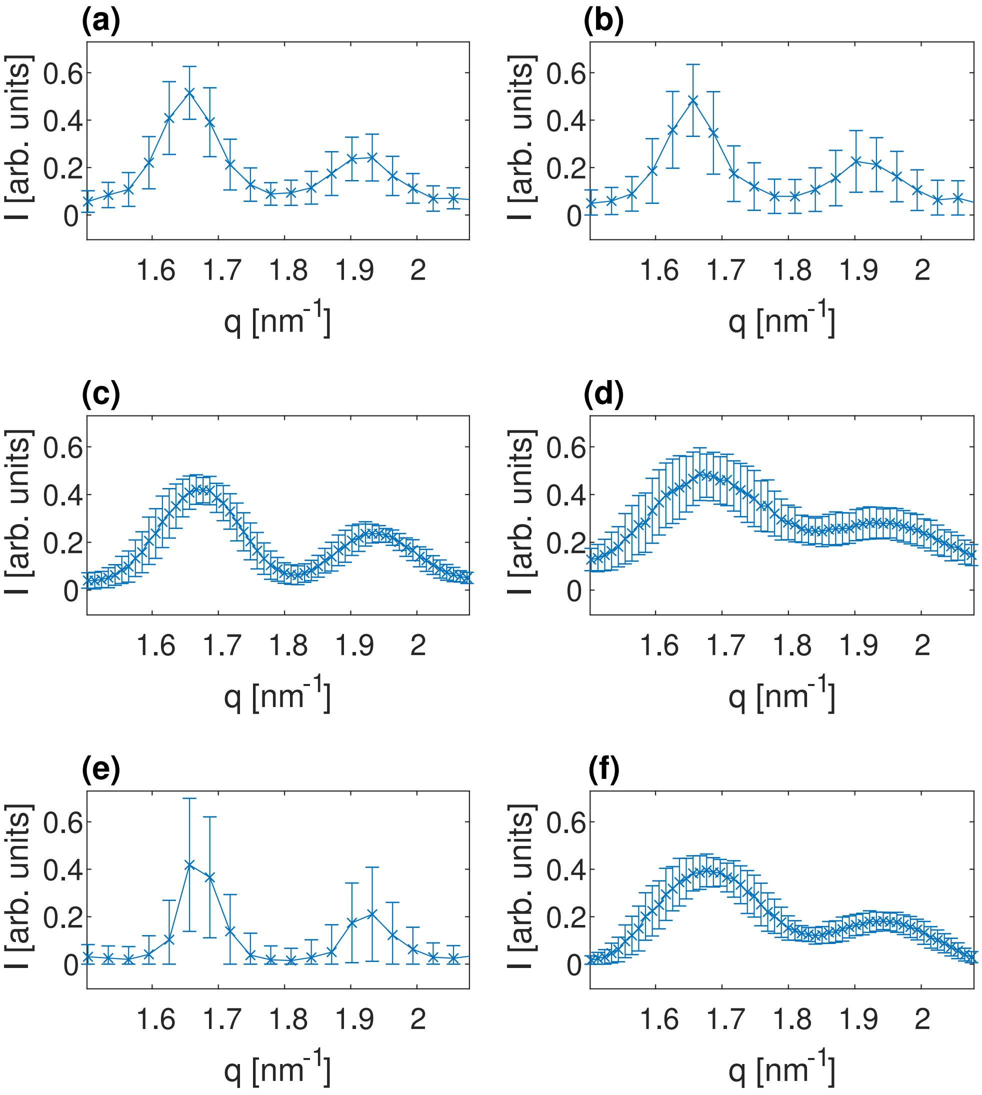

Figure S6: Re-plotting Figs. 9(g,h), 10(e) with error bar. Error bars are calculated from the intensity standard deviation at the 1D integration. for different retrieval approaches: (a) longest exposure time available with PSF of $0.6 \times 0.6\ mm^2$, the smallest used by all the algorithms, (b) similar exposure time as used by the SrSAXS algorithm, (c) SrSAXS algorithm, (d) SPS, (e) Lucy-Richardson algorithm and (f) full Farsiu algorithm.
